# O11 - Cor a 14: the missing link in the molecular diagnosis of hazelnut allergy?

**DOI:** 10.1186/2045-7022-4-S1-O11

**Published:** 2014-03-14

**Authors:** Margaretha Faber, Maaike De Graag, Catharina Van Der Heijden, Vito Sabato, Margo M Hagendorens, Chris H Bridts, Luc S De Clerck, Didier G Ebo

**Affiliations:** 1 Department of Immunology-Allergology-Rheumatology, Faculty of Medicine and Health Sciences, University of Antwerp, Antwerpen, Belgium; 2 Department of Immunology-Allergology-Rheumatology, Antwerp University Hospital, Antwerpen, Belgium; 3 Department of Pediatrics, Antwerp University Hospital, Antwerpen, Belgium

## Background

Hazelnut (Corylus avellana) allergy shows age and geographic related sensitization profiles that have not been fully resolved.

## Objectives

To study sensitization to hazelnut components in different age groups of hazelnut allergic patients and infants with atopic dermatitis (AD) sensitized to hazelnut in a birch-endemic region.

## Methods

Seventy-five hazelnut allergic patients, 14 infants below 1 year of age with AD sensitized to hazelnut and 15 age related healthy control individuals were tested for IgE reactivity to rCor a 1.04, rCor a 8, rCor a 9, nCor a 11, rCor a 14 by ImmunoCAP and rBet v 1 by ISAC 103 microarray.

## Results

Thirty-seven patients suffered from a systemic reaction and 38 patients reported an oral allergy syndrome (OAS) after eating hazelnut. In the population with systemic reactions, sensitization to Cor a 14 was seen in 19/20 preschoolchildren (median age (range) 2.6 years (1.0 – 5.4)), 8/10 schoolchildren (10.2 years (8.0 – 13.8 )) and 2/7 adults (28 years (18 – 33)) whereas sensitization to Cor a 9 was observed in 16/20 preschoolchildren, 7/10 schoolchildren and 3/7 adults. A minority of 13/37 and 5/37 was sensitized to Cor a 11 and Cor a 8. Combining of Cor a 14 and Cor a 9 enables us to correctly diagnose respectively 100 %, 80 % and 43 % of systemic reactions in preschool-, schoolchildren and adults. In contrast sensitization to Cor a 1.04 was generally associated with OAS, IgE reactivity to Cor a 1.04 was observed in respectively 6/7, 8/9 and 22/22 of preschool- , schoolchildren and adults. Sensitization to Cor a 14 was seen in two patients with OAS, although these sIgE levels to Cor a 14 were significantly lower. Twenty-one percent of the infants with AD showed Cor a 14 sensitization, whereas 4/14 and 1/14 showed IgE reactivity to Cor a 9 and Cor a 11.

## Conclusion

Quantification of Cor a 14 can be of great value in hazelnut allergy diagnosis. Sensitization to Cor a 14 predominantly occurs in pre- and schoolchildren with severe hazelnut allergy and can have early onset (< 1 year of age).

